# Neuro-fuzzy inference system Prediction of stability indices and Sodium absorption ratio in Lordegan rural drinking water resources in west Iran

**DOI:** 10.1016/j.dib.2018.02.075

**Published:** 2018-03-13

**Authors:** Afshin Takdastan, Majid Mirzabeygi (Radfard), Mahmood Yousefi, Abbas Abbasnia, Rouhollah Khodadadia, Hamed Soleimani, Amir Hossein Mahvi, Davood Jalili Naghan

**Affiliations:** a Department of Environmental Health Engineering, School of Health, Ahvaz Jundishapur University of Medical Sciences, Ahvaz, Iran; b Department of Environmental Health Engineering, School of Public Health, Tehran University of Medical Sciences, Tehran, Iran; c Student's Scientific Research Center, Tehran University of Medical Sciences, Tehran, Iran; d Ava Salamat Entrepreneurs Institute, Tehran, Iran; e Center for Solid Waste Research, Institute for Environmental Research, Tehran University of Medical Science, Tehran, Iran; f Deputy Health Sharekord University of Medical Science Health, Sharekord, Iran; g Environmental Technologies Research Center, Ahvaz Jundishapur University of Medical Science, Ahvaz, Iran

**Keywords:** Drinking water, Villages of Lordegan city, Stability index

## Abstract

According to World Health Organization guidelines, corrosion control is an important aspect of safe drinking-water supplies. Water always includes ingredients, dissolved gases and suspended materials. Although some of these water ingredients is indispensable for human beings, these elements more than permissible limits, could be endanger human health. The aim of this study is to assess physical and chemical parameters of drinking water in the rural areas of Lordegan city, also to determine corrosion indices. This cross-sectional study has carried out with 141 taken samples during 2017 with 13 parameters, which has been analyzed based on standard method and to estimate the water quality indices from groundwater using ANFIS. Also with regard to standard conditions, results of this paper are compared with Environmental Protection Agency and Iran national standards. Five indices, Ryznar Stability Index (RSI), Langlier Saturation Index (LSI), Larson-Skold Index (LS), Puckorius Scaling Index (PSI), and Aggressive Index (AI) programmed by using Microsoft Excel software. Owing to its simplicity, the program, can easily be used by researchers and operators. Parameters included Sulfate, Sodium, Chloride, and Electrical Conductivity respectively were 13.5, 28, 10.5, and 15% more than standard level. The amount of Nitrate, in 98% of cases were in permissible limits and about 2% were more than standard level. Result of presented research indicate that water is corrosive at 10.6%,89.4%,87.2%,59.6% and 14.9% of drinking water supply reservoirs, according to LSI, RSI, PSI, LS and AI, respectively.

**Specifications Table**

**Table:** 

Subject area	Chemistry
More specific subject area	Describe narrower subject area
Type of data	Tables, Figures
How data was acquired	This cross-sectional study has carried out with 141 taken samples during 2017 with 13 parameters, which has been analyzed based on standard method and to estimate the water quality indices from groundwater using ANFIS
Data format	Raw, Analyzed
Experimental factors	The mentioned parameters above, in abstract section, were analyzed according to the standards for water and wastewater treatment handbook.
Experimental features	The levels of physical and chemical parameters were determined.
Data source location	Chaharmahal and Bakhtiari province, Lordegan city Iran
Data accessibility	Data are included in this article

**Value of the data**

• Selecting the most realistic appropriate index, is one of the most sophisticated stages of water stability evaluations.
• The results of this study clearly indicated that with appropriate selection of input variables, ANFIS as a soft computing approach can estimate water quality indices properly and reliably.
• ANFIS approach is also extensile to solve, the other problems related to manage of the groundwater quality in agriculture and drinking water sectors such as nitrate, sulfate and etc.
• Considering present study, many of rural drinking water supply reservoirs, need to pay attention to achieve Iran national water quality standards.

## 1 Data

**Fig. 1:**
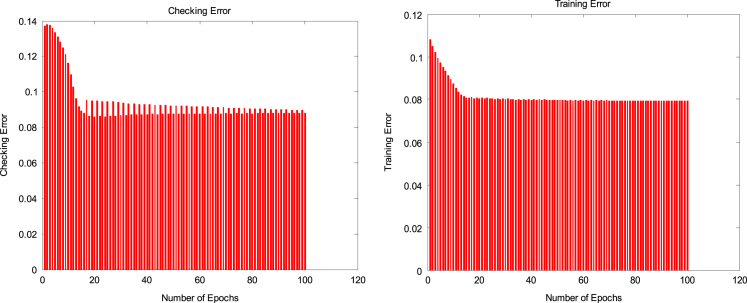
Checking and training errors (PSI index prediction) for optimization of epochs.

**Fig. 2:**
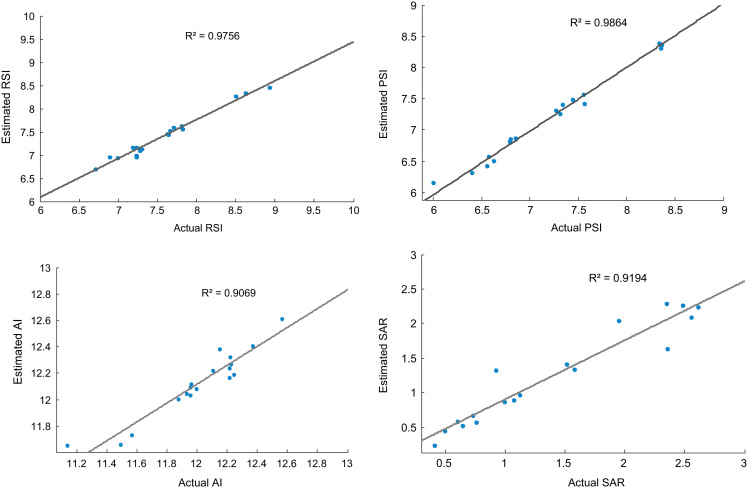
Estimated indexes versus real indexes for checking dataset.

**Fig. 3:**
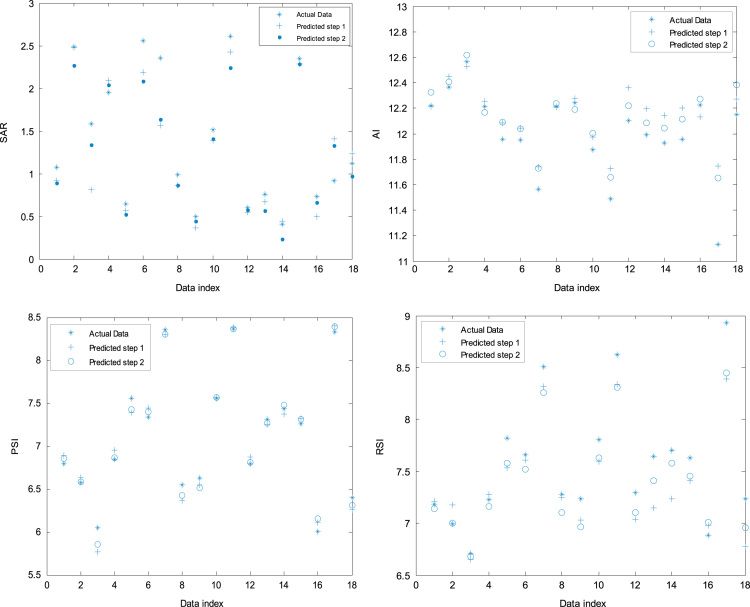
Comparison of first and second steps optimization in estimating of indexes for checking dataset.

**Fig. 4:**
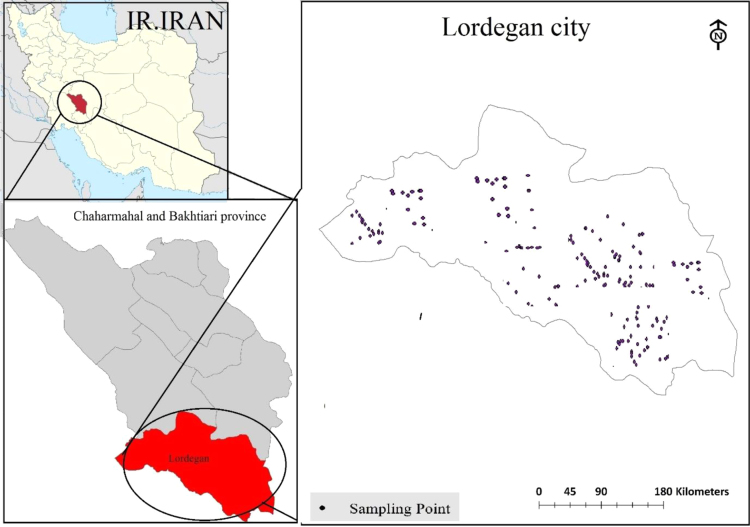
Location of water sampling sites in Lordegan city, Chaharmahal and Bakhtiari province.

The Table 1 shows Pearson's correlation factors between chemical water quality parameters and water indices and Table 2 shows predicting performance in different steps of ANFIS*,* Fig. 1. Checking and training errors (PSI index prediction) for optimization of epochs, Fig. 2 also shows estimated indexes versus real indexes for checking dataset, Fig. 3 shows comparison of first and second steps optimization in estimating of indexes for checking dataset and finally Fig. 4 shows location of water sampling sites in Lordegan city, Chaharmahal and Bakhtiari province.

**Table 1:** Pearson's correlation factors between chemical water quality parameters and water indices.

	TDS	TA	CH	PSI	RSI	AI	SAR
TDS	1						
TA	0.254467	1					
CH	0.413105	0.150231	1				
PSI	−0.38863	−0.7011	−0.74581	1			
RSI	−0.41141	−0.52671	−0.78821	0.941166	1		
AI	0.444762	0.478963	0.705398	−0.83255	−0.9527	1	
SAR	0.720472	0.111289	−0.03624	0.010696	−0.01206	0.10911	1

**Table 2:** Predicting performance in different steps of ANFIS.

Indexes	RMSE					
	Initial	1st step optimization	2nd step optimization	Radii	Epoch	CC
PSI						
- Train	0.1082	0.0875	0.0806	0.9	16	
- Check	0.1375	0.1164	0.0861			0.993
						
RSI						
- Train	0.2240	0.1839	0.1553	0.75	20	
- Check	0.2887	0.2808	0.2163			0.984
						
AI						
- Train	0.1363	0.1159	0.0969	0.6	100	
- Check	0.2284	0.2028	0.1673			0.952
						
SAR						
- Train	0.6474	0.6060	0.5473	0.55	20	
- Check	0.3284	0.3154	0.2713			0.9588

## 2 Experimental design, materials and methods

### 2.1 Study area description

Chaharmahal and Bakhtiari province is one of the 31 provinces of Iran. Lordegan is a city in Chaharmahal and Bakhtiari Province, Iran that coordinates is: 31°30′37″N 50°49′46″E.

### 2.2 Materials and methods

#### 2.2.1 Sample collection and analytical procedures

This cross-sectional study has carried out with 141 taken samples during 2017 with 13 parameters, which has been analyzed based on standard method [1], [2], [3], [4], [5], [6], [7], [8].

#### 2.2.2 Description of ANFIS

With introduction of fuzzy inference systems to the world of research, because of the great features of these methods in the control systems, as well as simple expression of the variables in terms of linguistic variables, use of this method in many applications was developed and expanded quickly. But designing a powerful fuzzy inference system, which needs to determine appropriate membership functions and fuzzy rules, is not easy, and mainly will be possible with a lot of trial and error and with the involvement of experts' comments in order to achieve the best performance, and in some cases, identification of all the rules is impossible. For this reason, the idea of using learning algorithms of the artificial neural network was introduced for fuzzy systems. ANFIS is an adaptive neural fuzzy inference system which was first proposed in 1993 by Jung. The system is an intelligent hybrid method, which uses features of the Takagi-Sugeno fuzzy inference system and adaptive neural network [9], [10], [11]. ANFIS, using artificial neural network learning algorithms, including gradient descent algorithm and back-propagation algorithm leads to improve fuzzy system rules. ANFIS, because of having the adaptive fuzzy system parameters compared with artificial neural network, is trained faster, and usually provides better results. Schematic structure of ANFIS, with two input variables and an output variable, which lead to the Sugeno model, is shown in Fig. 1. Two rules (Eqs. (1), (2)) in the Sugeno fuzzy model can be written as follows:

(1) IfxisA1andyisB1thenf1=p1x+q1+r1

(2) IfxisA2andyisB2thenf2=p2x+q2+r2

Generally, the structure of an ANFIS model has 5 distinct layers, and each of these layers plays a special role. In the first layer, every node i, according to Eq. (3), is known as a comparative node, and the outputs of this layer represent the degree of membership assigned to inputs in the fuzzy form.

(3) $$O^{3}i=\mu \;Ai(x)$$

In the above equation, x is an input variable to node i and Ai is small, large… linguistic labels, that are associated with the node function. In other words, O^3^ is membership function of fuzzy set A, which the input variable x has certain circumstances in it. Membership function A, can be Gaussian function or triangular membership function and so on. The membership function parameters that are used in this layer are known as the first parameter. In MATLAB, using genfis1 and genfis2functions, you can create a fuzzy inference system, which covers the first layer of ANFIS [9], [12]. In the genfis1 function, to create a fuzzy system, there is to need to specify the number and type of membership function well. However, genfis2 function creates this fuzzy system using Subtractive clustering. In this study, genfis2 function is used with Gaussian membership function and Subtractive clustering to create the fuzzy system. In the Subtractive clustering, it is assumed that all data have candidate for clustering center according to the density of the surrounding data. So, using the following Eq. (4), we can have a measure of density of data xi.

(4) $$\mathrm{Di}=\sum_{j=1}^{n}\exp\left[\frac{\left\|\mathrm{Xi}-\mathrm{Xj}\right\|2}{({\mathrm{ra}/2)}^{2}}\right]$$

Here, ra is a positive constant, which indicates the radius of the neighbored radius. Therefore, a data with the greatest amount of density (Di), has more the number of data points which are located in the surrounding neighborhood. First cluster center Xc1 is dedicated to data that has the highest density Dc1. The density value of each data point xi is modified as follows Eq. (5) [9], [10], [11], [12].

(5) $$\mathrm{Di}=\mathrm{Di}-\mathrm{Dc}1\exp\left[-\frac{{\left\|\mathrm{Xi}-\mathrm{Xc}1\right\|}^{2}}{({\frac{\mathrm{rb}}{2})}^{2}}\right]$$

Here, rb is a positive constant, and defines a neighborhood, which has a significant reduction in the amount of density. Therefore, the numbers of data points that are located near the center of the first cluster xc1 significantly reduce the amount of density. After correcting the density function, the next cluster center is belonged to a data which has maximum density. This process continues until a sufficient number of clusters are reached. In the second layer of ANFIS, the outputs of the first layer are combined. As a result, the output of this layer can be written according to Eq. (6).

(6) $$O_{1}^{6}=W_{1}=\mu_{\mathrm{Ai}}\;(X)\;\mu_{\mathrm{Bi}}\;(y)$$

Here, each output node burning represents the firing strength of a rule. The next layer is the second layer, which normalizes the output of before layers output, and calculates the firing strength of i-th rule than the firing strength of all the rules based on Eq. (7).

(7) $$O_{1}^{7}=W_{i}=\;\frac{Wi}{W1+W2}$$

Nodes of the fourth layer have the adaptive nature, and the parameters of this node (ri, qi, pi) are called as consequent parameters. Eq. (8) can be written to describe the layer 4. In this equation, f is a first order function of the Sugeno fuzzy system.

(8) $$O_{i}^{8}=W_{i}f_{i}=\mathrm{Wi}\left(p_{i}x+q_{i}y+r_{i}\right)$$

And finally, for the output of layer 5, which is the final output of the ANFIS system, we can write Eq. (9) [9], [10], [11], [12].

(9) $$O_{i}^{5}=z=\Sigma i\;\bar{w}ifi$$
